# NF-κB Mediates Tumor Necrosis Factor α-Induced Expression of Optineurin, a Negative Regulator of NF-κB

**DOI:** 10.1371/journal.pone.0005114

**Published:** 2009-04-02

**Authors:** Cherukuri Sudhakar, Ananthamurthy Nagabhushana, Nishant Jain, Ghanshyam Swarup

**Affiliations:** Centre for Cellular and Molecular Biology, Council of Scientific and Industrial Research, Hyderabad, India; Johns Hopkins School of Medicine, United States of America

## Abstract

Optineurin is a ubiquitously expressed multifunctional cytoplasmic protein encoded by *OPTN* gene. The expression of optineurin is induced by various cytokines. Here we have investigated the molecular mechanisms which regulate optineurin gene expression and the relationship between optineurin and nuclear factor κB (NF-κB). We cloned and characterized human optineurin promoter. Optineurin promoter was activated upon treatment of HeLa and A549 cells with tumor necrosis factor α (TNFα). Mutation of a putative NF-κB-binding site present in the core promoter resulted in loss of basal as well as TNFα-induced activity. Overexpression of p65 subunit of NF-κB activated this promoter through NF-κB site. Oligonucleotides corresponding to this putative NF-κB-binding site showed binding to NF-κB. TNFα-induced optineurin promoter activity was inhibited by expression of inhibitor of NF-κB (IκBα) super-repressor. Blocking of NF-κB activation resulted in inhibition of TNFα-induced optineurin gene expression. Overexpressed optineurin partly inhibited TNFα-induced NF-κB activation in Hela cells. Downregulation of optineurin by shRNA resulted in an increase in TNFα-induced as well as basal NF-κB activity. These results show that optineurin promoter activity and gene expression are regulated by NF-κB pathway in response to TNFα. In addition these results suggest that there is a negative feedback loop in which TNFα-induced NF-κB activity mediates expression of optineurin, which itself functions as a negative regulator of NF-κB.

## Introduction

Optineurin is a cytoplasmic protein that is ubiquitously expressed although it shows higher level of expression in retina, brain, heart, skeletal muscle, placenta and kidney [1]–[4]. Mutations in optineurin are associated with certain glaucomas, a group of eye diseases that cause blindness [5]–[14]. Optineurin interacts with several proteins such as Rab8, Huntingtin, myosin VI, RIP, transcription factor IIIA, metabotropic glutamate receptor, TBK1 etc [1], [15]–[20]. Based on interaction with myosin VI, its role in vesicular trafficking between Golgi and plasma membrane has been proposed [17]. Recently it has been shown that optineurin negatively regulates TNFα-induced NF-κB activation by binding to polyubiquitinated RIP (19). Towards the C-terminal end, a ubiquitin-binding domain has been identified in optineurin, which is also present in NEMO, a sub-unit of protein kinase IKK complex involved in NF-κB regulation [19]. Optineurin plays a role in the regulation of expression of many genes [21] although the mechanisms involved are yet to be elucidated. Optineurin gene and protein expression is induced by treatment of cells with TNFα and interferons [1], [16]. However the mechanisms by which these cytokines activate optineurin gene expression are not known.

TNFα is a cytokine that plays an important role in inflammation, immune response, regulation of cell death, cell proliferation and cancer [22], [23]. The biological effects of TNFα are mediated by its binding to trimeric receptor, TNFR-1 which results in activation of two important signalling pathways that lead to activation of transcription factor NF-κB and caspase-8. NF-κB comprises a family of inducible transcription factors that serve as important regulators of host immune response and inflammatory response [22], [23]. NF-κB is also involved in protecting cells from apoptosis by inducing many anti-apoptotic genes. NF-κB activity is regulated through association with an inhibitor, IκB which keeps NF-κB in the cytoplasm [22], [23]. NF-κB is activated in the trabecular meshwork cells in glaucoma where it is involved in cytoprotection in response to oxidative stress [24].

Overexpressed optineurin provides protection to NIH 3T3 cells against cell death induced by high level of oxidative stress [4]. Recently we have shown that overexpression of normal optineurin sensitizes RGC-5 cells to TNFα-induced cell death whereas in HeLa cells it reduces TNFα-induced cell death [25]. It is likely that altered level of endogenous optineurin may affect survival of cells under certain conditions of stress. It has been suggested that reduction in expression of optineurin due to mutation in the coding region, rather than altered function may contribute to the development of glaucoma [19]. Therefore it is important to understand the molecular mechanisms which regulate the level of optineurin.

Here we have investigated the molecular mechanisms which regulate optineurin gene expression, and also studied the relationship between optineurin and NF-κB. We have cloned and characterized human optineurin promoter. The results presented here show that optineurin promoter activity induced by TNFα is regulated by NF-κB, which itself is in turn, negatively regulated by optineurin.

## Results

**Figure 1:**
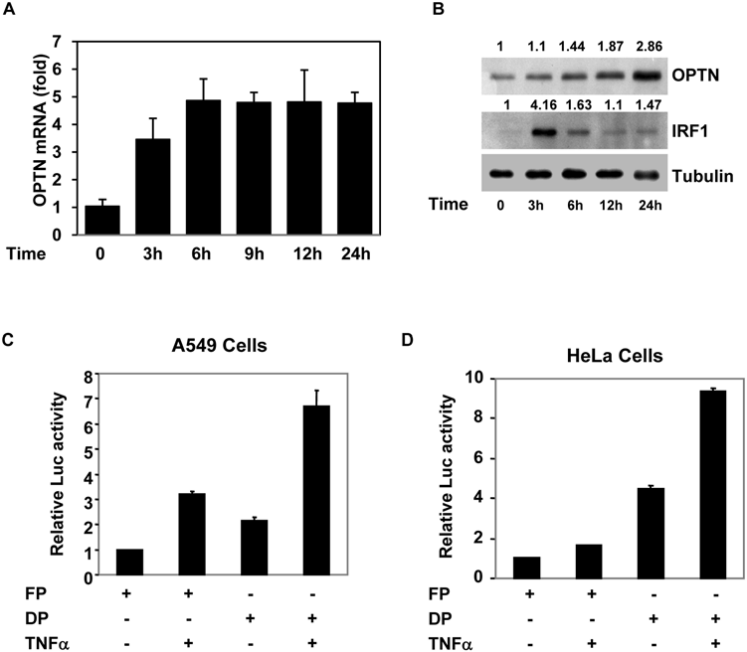
Induction of optineurin gene expression and promoter activation by TNFα. (A), A549 cells were treated with TNFα (10 ng/ml) for indicated time (3–24 hours). Total RNA was then isolated and the level of optineurin mRNA was determined by real time RT-PCR. GAPDH was used as a control. (B) Effect of TNFα on optineurin protein level. A549 cells were treated with TNFα (10 ng/ml) for indicated time. The cell lysates were then prepared for immunoblotting which was performed using antibodies against optineurin, IRF-1 and tubulin (loading control). The numbers at the top indicate relative amount of protein. Activation of optineurin promoter by TNFα in A549 cells (C) or HeLa cells (D). Cells grown in 24 well plates were transfected with 100 ng of optineurin promoter-reporter plasmid (full length construct pGL-FP or deletion construct pGL-DP) along with pCMV.SPORT β-gal plasmid. After 6 hours of transfection TNFα was added (10 ng/ml). After another 18 hours cell lysates were prepared for reporter assays. Luciferase activities relative to untreated control (taken as 1.0) are shown (n = 3) after normalizing with β-galactosidase enzyme activities.

TNFα has been shown to induce optineurin gene and protein expression in HeLa and some other cells [1], [16]. To understand the molecular mechanisms involved in TNFα-induced optineurin gene expression we used human lung carcinoma cell line A549 which is responsive to TNFα. Initially we confirmed that TNFα treatment increases optineurin gene expression in A549 cells in a time-dependent manner as determined by real time RT-PCR analysis (Fig. 1A). The optineurin mRNA level increased by 3 hours of TNFα treatment reaching maximum level after 6 hours. The optineurin mRNA level remained at increased level even after 24 hours of treatment with TNFα. The level of optineurin protein increased gradually upon TNFα treatment and reached high level after 24 hours (Fig. 1B). In contrast IRF-1 protein level showed a transient increase after 3 hours of TNFα treatment and declined thereafter (Fig. 1B).

### TNFα activates optineurin promoter

We cloned about 1 kb of DNA sequence upstream of human optineurin cDNA sequence by designing appropriate primers. This putative optineurin promoter sequence matched completely with the sequence reported in human genome data base. This putative promoter was cloned in pGL-3 promoterless vector upstream of luciferase reporter gene. This promoter-reporter plasmid (FP) was transfected in HeLa cells and after 24 h these cells were lysed for reporter assays. This promoter showed 147-fold higher activity than the promoterless vector (figure not shown). The activity of this promoter was increased by 2.3-fold upon treatment of cells with TNFα in A549 cells (Fig. 1C) and by 60% in HeLa cells (Fig. 1D).

**Figure 2:**
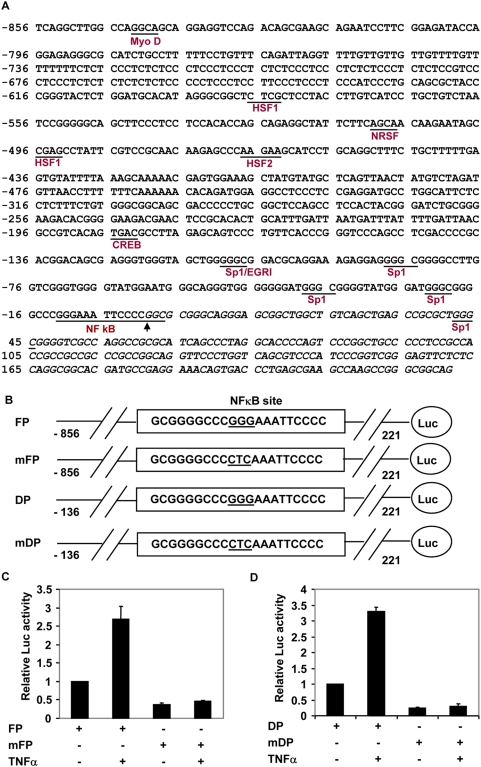
Effect of mutation of putative NF-κB site in optineurin promoter on TNFα-induced activity. (A) Nucleotide sequence of human optineurin promoter. Putative transcription factor binding sites for various transcription factors such as Sp1, NF-κB, MyoD, HSF1, HSF2, NRSF and CREB are shown. The 5′ mRNA (exon 1) sequence is shown in italics. First nucleotide of optineurin mRNA is taken as transcription start site (+1), indicated by an arrow. (B) Schematic representation of various promoter reporter constructs used for experiments in C, D. Full length promoter (FP), deleted promoter (DP) and their NF-κB site mutants mFP and mDP are shown. The nucleotide sequence of putative NF-κB site is shown and the 3 nucleotides which were mutated are underlined. (C) Mutant or normal full length optineurin promoter constructs (mFP or FP) were transfected in A549 cells and after 6 hours of transfection TNFα was added. Cell lysates were prepared after 24 hours of transfection for reporter assays. Luciferase activities relative to untreated control promoter (taken as 1.0) are shown (n = 3). (D), The same experiment as in (C) was carried out using deleted promoter, DP, or its mutant, mDP.

The nucleotide sequence of this 1077 bp putative promoter, which includes 221 bp of exon-1, is shown in Figure 2A. There are 4 splice variants reported for optineurin mRNA in the database which differ in their 5′-untranslated region but code for the same 577 amino acid protein (Accession Nos. NM_001008211, NM_001008212, NM_001008213 and NM_021980). Exon-1 is present in all these 4 variants. The beginning of exon-1 is denoted as +1. Analysis of this promoter showed several putative Sp1 sites and one NF-κB site located immediately upstream of transcription start site (Fig. 2A). There is no TATA box or initiator element present in this promoter. Putative binding sites for heat shock factors, HSF1 and HSF2, MyoD, neuron-restrictive silencing factor (NRSF, also known as REST) and cyclic AMP response element binding protein (CREB) were also identified. A smaller promoter was made which contained putative Sp1 sites and the NF-κB site (−136 to +221; named DP). This smaller promoter was activated by TNFα as the full length promoter in A549 and HeLa cells (Fig. 1C, D). The smaller promoter showed more basal activity than the full length promoter in A549 as well as in HeLa cells (Fig. 1C, D). These results showed that the DNA sequence elements which mediate TNFα-induced as well as basal promoter activity are present in the small promoter. In addition these results suggest that a negative regulatory element is present in sequences upstream of the minimal promoter.

### Role of NF-κB in optineurin promoter activation

One of the important signalling pathways induced by TNFα results in the activation of transcription factor NF-κB. Therefore we mutated the putative NF-κB site in optineurin promoter and analysed the effect of this mutation on reporter activity. Three nucleotides in the core sequence of putative NF-κB binding site were mutated (GGG changed to CTC, Fig. 2B). Mutation of NF-κB site in full length as well as minimal promoter resulted in nearly complete loss of activation by TNFα in A549 cells (Fig. 2C, D). In addition, basal promoter activity was also significantly reduced in full length as well as minimal promoter (Fig. 2C, D). These results showed that NF-κB site is required for activation of optineurin promoter by TNFα. In addition our results suggest that NF-κB site is required for basal promoter activity.

**Figure 3:**
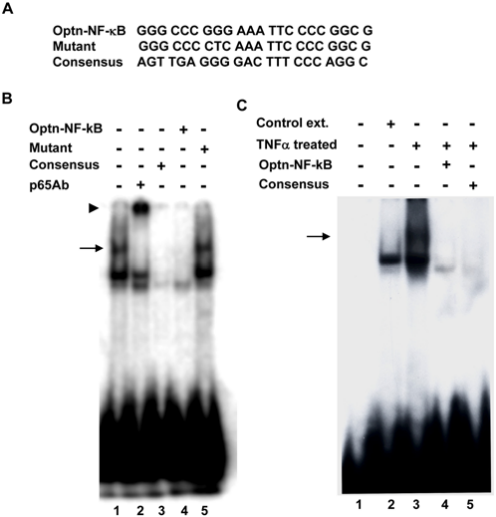
Electrophoretic mobility shift assay. (A), Nucleotide sequence of synthetic oligonucleotide corresponding to putative NF-κB site in optineurin promoter is shown which is used for assay (Optn. NF-κB). A mutant of this oligonucleotide (mutant) and a consensus NF-κB oligonucleotide used for competition are also shown. (B) EMSA was carried out using radiolabelled oligonucleotide with nuclear extracts from A549 cell treated with TNFα for 15 minutes. A 50-fold excess of unlabeled self (lane 4), mutant (lane 5) or consensus NF-κB (lane 3) oligonucleotide was used for competition. The nuclear extract was preincubated with p65 NF-κB antibody for 30 min at 37°C before adding the labelled probe (lane 2). Arrow indicates gel shift. Arrowhead indicates super shift. (C) EMSA was carried out as in panel B except that control extract was also used (lane 2).

A synthetic oligonucleotide corresponding to putative NF-κB site present in the optineurin promoter was used for gel shift assay (Fig. 3A). The oligonucleotide was labelled with ^32^P and incubated with nuclear extract prepared from A549 cells treated with TNFα for 15 min. Binding to this oligonucleotide was competed out with 50-fold excess of unlabeled self oligonucleotide and also with consensus NF-κB-binding oligonucleotide (Fig. 3B) but not by a mutant oligonucleotide in which NF-κB site was inactivated by substitution of 3 nucleotides (Fig. 3B). Preincubation of the nuclear extract with p65 NF-κB antibody resulted in the super shift of the band. Gel shift assay was also carried out with nuclear extract from control untreated cells which did not show the upper band shifted by p65 antibody (Figure 3C). These results along with mutational analysis of the promoter show that a functional NF-κB-binding site is present in optineurin promoter.

**Figure 4:**
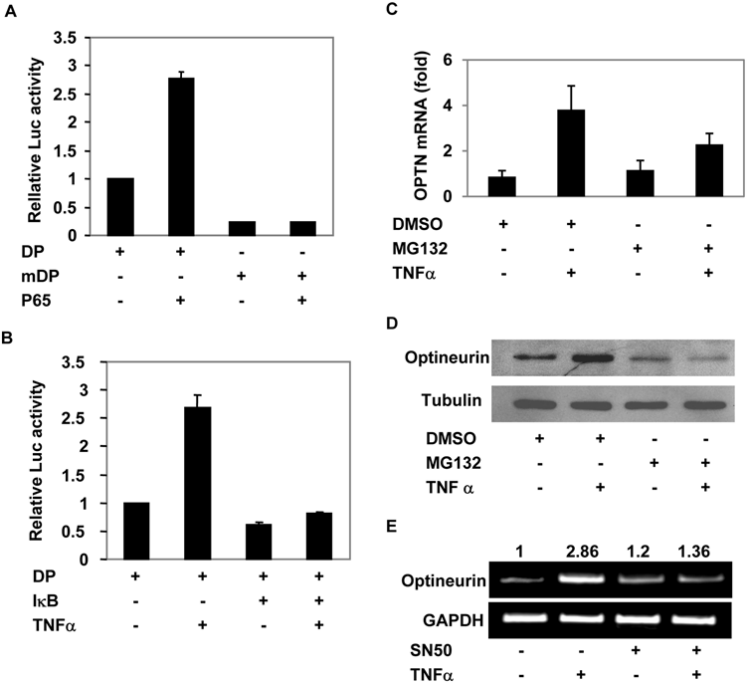
NF-κB mediates optineurin gene expression. (A) NF-κB p65 activates optineurin promoter through NF-κB site. Optineurin minimal promoter constructs pGL-DP or pGL-mDP (100 ng) were transfected without or with NF-κB p65 expression plasmid (100 ng) in A549 cells. After 24 hours of transfection cell lysates were prepared for reporter assays. Luciferase activities relative to control pGL-DP (taken as 1.0) are shown (n = 3). (B) IκBα inhibits TNFα-induced NF-κB activity. pGL-DP was transfected without or with IκBα super repressor expression plasmid (100 ng). TNFα was added 6 hours after transfection. Luciferase activities relative to control are shown (n = 3). (C) Blocking of NF-κB activation inhibits TNFα-induced optineurin gene expression. A549 cells were preincubated with 25 µM MG132 or solvent DMSO (0.1%) for 30 minutes prior to treatment with TNFα. After 6 hours of treatment with TNFα, RNA was isolated and the level of optineurin mRNA was determined by real time RT-PCR analysis. GAPDH was used as a control. (D) A549 cells were treated with MG132 as in panel C and after 6 hours of treatment with TNFα cell lysates were subjected to Western blotting. (E) A549 cells were treated with 100 µg/ml of SN-50 peptide for 30 minutes prior to treatment with TNFα for 6 hours. The picture shows RT-PCR analysis for optineurin gene expression.

The gel shift assay showed that p65 was able to bind NF-κB site present in the optineurin promoter. To further analyse the role of p65 NF-κB, A549 cells were transfected with optineurin promoter-reporter plasmid with or without p65 expression plasmid. Coexpression of p65 resulted in an increase in promoter activity but the mutant promoter (in which NF-κB site was abrogated) was not activated by p65 (Fig. 4A).

### IκB inhibits optineurin promoter activity

In resting cells most of the NF-κB remains inactive due to its sequestration in the cytoplasm by a group of inhibitory proteins known as inhibitors of κB (IκB). Upon stimulation by a cytokine like TNFα, IκBs are rapidly phosphorylated and then degraded by ubiquitin proteasome pathway resulting in the nuclear translocation of NF-κB. Since NF-κB activation depends on IκB, we looked into the effect of IκB on optineurin promoter activity. For this purpose we used a super-repressor form of IκBα in which two serine residues (S32, S36) are mutated so that it is not phosphorylated or degraded. Optineurin promoter reporter plasmids were transfected with or without IκBα super-repressor plasmid in A549 cells. After 6 hours of transfection TNFα was added. TNFα increased the promoter activity by 2.6 fold. Overexpression of IκBα super-repressor abrogated the TNF-α induced activation of the promoter. Basal activity of the promoter was also reduced upon coexpression of IκBα (Fig. 4B). These results suggest that optineurin promoter activity is regulated by IκB-NF-κB pathway in response to TNFα.

### NF-κB activation is required for TNFα-induced optineurin gene expression

Our experiments described so far suggested that NF-κB plays an important role in optineurin promoter activation by TNFα. We next examined the role of NF-κB in optineurin gene expression in response to TNFα. For this purpose we used proteasome inhibitor MG132 which inhibits NF-κB activation by preventing degradation of IκB [26]. A549 cells were pretreated with MG132 for 30 min and then treated with TNFα for 6 hours. Blocking of NF-κB activation by MG132 resulted in inhibition of TNFα-induced optineurin gene expression as determined by real time RT-PCR analysis (Fig. 4C). Blocking of NF-κB activation also resulted in reduced level of TNFα-induced optineurin protein level (Fig. 4D). We also used NF-κB inhibitor SN-50 to check its effect on TNFα-induced optineurin gene expression. Pretreatment of A549 cells with SN-50 resulted in inhibition of TNFα-induced optineurin gene expression (Fig. 4E).

### Optineurin and E50K mutant inhibit activation of NF-κB

**Figure 5:**
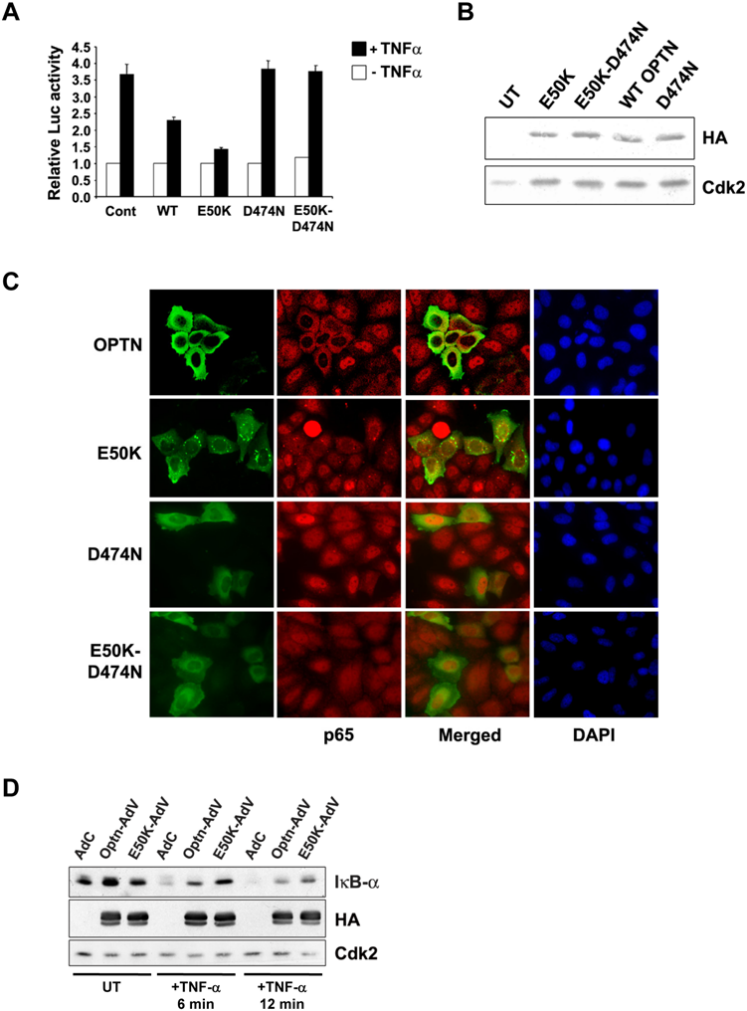
Optineurin and E50K mutant inhibit TNFα-induced NF-κB activity. (A) NF-κB-Luc reporter plasmid (25 ng) was transfected without or with optineurin expression plasmid (100 ng) in HeLa cells. After 22 hours of transfection cells were treated with TNFα for 4 hours. Luciferase activities relative to untreated control are shown (n = 3). (B) Western blot showing expression of optineurin and its mutants using HA tag antibody. Cdk2 was used as control. (C) Optineurin and E50K mutant inhibit TNFα-induced nuclear translocation of NF-κB p65. HeLa cells grown on coverslips were transfected with optineurin expression plasmid. After 24 hours of transfection cells were treated with TNFα for 30 min. The cells were then fixed and stained for optineurin (HA tag, FITC green) and p65 (Cy3, red) and visualized using a fluorescence microscope. (D) HeLa cells were infected with adenoviruses for expressing optineurin (Optn-AdV), its E50K mutant (E50K-AdV) or control virus (AdC). After 36 hours of infection, the cells were treated with TNFα for 6 min or 12 min or left untreated. Cell lysates were then prepared for western blotting with antibodies for IκBα, HA tag and Cdk2.

Various lines of evidence suggest that NF-κB is a regulator of optineurin gene expression. Some targets of NF-κB are known to regulate NF-κB activity in signaling pathways [22], [27]. Therefore we examined the role of optineurin in the modulation of TNFα-induced NF-κB activation. The effect of ectopic expression of wild type optineurin and its E50K mutant on TNFα-induced NF-κB activation was determined. HeLa cells were transfected with NF-κB luciferase reporter construct along with or without optineurin expression plasmids. After 22 hours of transfection these cells were treated with TNFα for 4 hours. Overexpression of optineurin or E50K mutant did not affect the basal NF-κB reporter activity. In response to TNFα the NF-κB reporter was activated to 3.7 fold. This TNFα-induced NF-κB reporter activity was partially inhibited by optineurin (Fig. 5A). These observations suggest that optineurin negatively regulates TNFα-induced NF-κB activation. The inhibition of NF-kB activation by E50K mutant was significantly more (P<0.05) than that observed with wild type optineurin. This was not due to higher level of expression of mutant optineurin (Figure 5B). To further validate these observations, effect of overexpression of optineurin and its E50K mutant on TNFα-induced nuclear translocation of NF-κB p65 was investigated. HeLa cells grown on coverslips were transfected with a plasmid expressing HA-tagged optineurin, and after 24 hours these cells were treated with TNFα for 30 min. The cells were then stained for optineurin (HA antibody) and NF-κB p65. Microscopic examination of cells revealed that nuclear translocation of NF-κB p65 was inhibited in most of the cells expressing exogenous optineurin and its E50K mutant (Fig. 5C).

TNFα stimulus results in rapid phosphorylation and proteasome mediated degradation of IκB proteins, resulting in nuclear translocation of NF-κB subunits where they regulate the expression of target genes. HeLa cells were infected with adenoviruses expressing wild type or E50K mutant optineurin and were treated with TNFα for 6 min and 12 min. A significant amount of IκB-α was degraded by 6 min of TNFα stimulation and by 12 min it was almost completely degraded. In cells overexpressing E50K mutant, substantial levels of IκB-α persisted even after 12 min of TNFα treatment suggesting that E50K mutant inhibits TNFα-induced IκB-α degradation (Fig. 5D). IκB-α levels were higher in TNFα-treated cells expressing E50K mutant compared to those expressing wild type optineurin. These results suggest that E50K mutant optineurin inhibits TNFα-induced NF-κB activation by possibly affecting an upstream event.

### Ubiquitin-binding region is essential for inhibition of NF-κB activation by E50K mutant

A recent study has shown that optineurin binds lysine 63-linked polyubiquitin chains, and competes with NEMO for the binding of ubiquitinated RIP. A mutation in the conserved ubiquitin binding region (D474N) abolishes ubiquitin binding ability of optineurin and NF-κB activation [19]. Since E50K mutant optineurin inhibits TNFα-induced NF-κB activation more strongly than wild type optineurin, a mutant of optineurin containing both E50K and D474N mutations was generated to gain further insights into the regulation of TNFα-induced NF-κB signaling by mutant optineurin. HeLa cells were transfected with D474N or E50K or E50K-D474N mutants along with NF-κB luciferase reporter. As expected the D474N mutant optineurin did not inhibit TNFα-induced NF-κB activation. However, the E50K-D474N double mutant also did not inhibit TNFα- induced NF-κB activation (Fig. 5A). The expression of mutants was confirmed by Western blotting (Fig. 5B). To further validate these observations, sub-cellular distribution of p65 subunit of NF-κB was studied. HeLa cells were transfected with either D474N or E50K-D474N mutants and 24 hrs after transfection, cells were treated with TNFα for 30 min. Immunostaining of p65 showed that in response to TNFα, p65 translocates to nucleus in cells expressing D474N mutant and in those expressing E50K-D474N mutant optineurin (Fig. 5C). These observations suggest that the inhibition of NF-κB activation by E50K mutant requires a functional ubiquitin binding region.

### Downregulation of optineurin activates NF-κB

**Figure 6:**
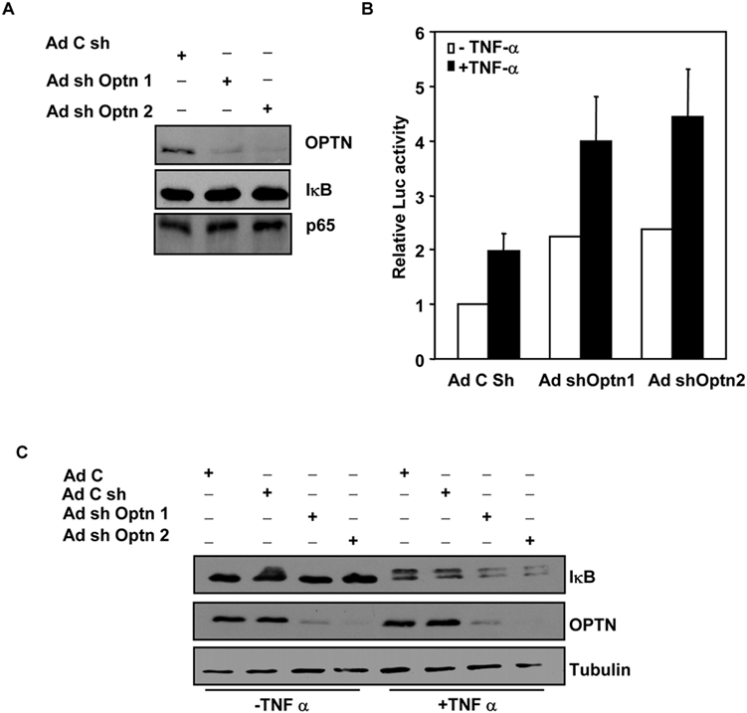
Knockdown of optineurin by shRNA enhances TNFα-induced NF-κB activity. (A) HeLa cells were infected with Ad-shOptn1, Ad-shOptn2 or control viruses and after 72 hours cell lysates were subjected to western blotting using antibodies for optineurin, IκBα and p65 NF-κB. Control virus (AdC sh) expresses shRNA of unrelated sequence of same length. (B) HeLa cells were infected with Ad-shOptn1, Ad-shOptn2 or control viruses. After 48 h of infection these cells were transfected with NF-κB reporter plasmid (25 ng) along with β-galactosidase expression plasmid. After 22 hours of transfection the cells were treated with TNFα (10 ng/ml) for 4 hours. Cell lysates were then made for reporter assays. The data represent luciferase activities relative to untreated control taken as 1.0 (n = 3). (C) HeLa cells were infected with indicated adenoviruses and after 72 hours treated with TNFα for 6 min or left untreated. Cell lysates were subjected to western blot analysis using antibodies for IκB, optineurin and tubulin (loading control). AdC, control virus not expressing any shRNA.

Since overexpression of optineurin inhibited TNFα-induced NF-κB activity, we determined the effect of downregulation of endogenous optineurin on basal and TNFα-induced NF-κB activity. For this purpose two shRNAs were expressed which target two different regions of optineurin mRNA. Infection of HeLa cells with adenoviruses expressing shRNAs (Ad-shOptn1 and Ad-shOptn2) resulted in significant decrease in optineurin protein level after 72 hours of infection, as determined by western blotting (Fig. 6A); however there was no significant change in the level of IκBα or NF-κB p65. A virus expressing shRNA of unrelated sequence of same length was used as a control. To determine the effect of downregulation of optineurin on NF-κB activity, HeLa cells were infected with Ad-shOptn1, Ad-shOptn2 or control viruses. After 48 h of infection these cells were transfected with NF-κB reporter plasmid along with β-galactosidase expression plasmid. After another 22 hours, these cells were treated with TNFα for 4 hours. Downregulation of optineurin by both shRNAs resulted in significant increase (P<0.05) in basal as well as TNFα-induced NF-κB activity (Fig. 6A, B). To determine the effect of optineurin knockdown upon IκBα levels, HeLa cells were infected with the required adenoviruses and after 72 hours treated with TNFα for 6 min. Western blot analysis showed that downregulation of optineurin by both shRNAs resulted in enhanced degradation of IκB upon TNFα stimulation (Fig. 6C). These results suggest that endogenous optineurin inhibits TNFα-induced NF-κB activation and this inhibition occurs at a step upstream of IκB degradation.

## Discussion

To begin to understand the molecular mechanisms which regulate optineurin gene expression we have cloned and characterized optineurin gene promoter. This promoter is active in HeLa as well A549 cells and is activated upon treatment of cells by TNFα. This promoter does not have a TATA box or an initiator element. It showed several putative Sp1 sites and one NF-κB site. Mutation of NF-κB site resulted in loss of activation of promoter by TNFα. In addition basal promoter activity was also reduced. Overexpression of IκBα resulted in inhibition of basal as well as TNFα-induced promoter activity. These results show that NF-κB is a crucial regulator of optineurin promoter activity. The location of NF-κB site immediately upstream of exon-1, requirement of NF-κB site for basal promoter activity and lack of an initiator element lead us to speculate that NF-κB might be involved in initiation of transcription from this promoter. Activation of optineurin promoter by TNFα suggests that TNFα-induced induction of optineurin gene expression is likely to be due to the activation of the promoter. A recent study has identified several target genes of NF-κB including optineurin [28].

Deletion analysis resulted in identification of a minimal or core promoter (−136 to +221) which is activated by TNFα and shows higher basal activity than the longer promoter in both HeLa as well as A549 cells. Higher basal activity of smaller promoter suggests that negative regulatory elements are present between −856 and −136 region of the promoter. However the negative regulatory elements present between −856 and −136 region do not seem to play a role in TNFα-induced activation because the core promoter shows as much activation by TNFα as the longer promoter. Presence of a putative MyoD-binding site in optineurin promoter might explain high level of optineurin mRNA reported in skeletal muscle from human and monkey [1], [2].

The NF-κB activity is increased in the trabecular meshwork cells obtained from the eyes of glaucomatous patients of diverse etiology [24]. Cells of the trabecular meshwork regulate aqueous outflow which controls intraocular pressure. It was shown that enhanced NF-κB activity, due to higher level of interleukin-1, protects glaucomatous trabecular meshwork cells from apoptosis induced by oxidative stress [24]. NF-κB p50-deficient mice develop glaucoma-like optic neuropathy [29]. Thus NF-κB has cytoprotective function in various tissues of the eye. Recently it has been shown that over-expressed optineurin protects NIH 3T3 cells from apoptosis induced by oxidative stress [4]. Whether NF-κB-induced optineurin contributes to cytoprotection against oxidative stress, is yet to be investigated.

Recently it has been shown that optineurin negatively regulates basal as well as TNFα-induced NF-κB activity in some cell lines like HeLa S3 and Hek293 [19]. Our results show that optineurin inhibits TNFα-induced NF-κB activity in HeLa cells although basal activity was not inhibited. However downregulation of endogenous optineurin by shRNA resulted in upregulation of basal as well as TNFα-induced NF-κB activity. It has been suggested that TNFα-induced NF-κB activity is inhibited by optineurin due to its competition with NEMO for binding to ubiquitinated RIP [19]. The mechanism by which optineurin negatively regulates basal NF-κB activity is yet to be determined. A glaucoma-associated mutant of optineurin, E50K, showed significantly more inhibition of TNFα-induced NF-κB activation in HeLa cells. However this may not have any relevance to glaucoma.

NF-κB activation is associated with several diseases such as chronic inflammation, cancer and glaucoma [24], [30], [31]. Induction of optineurin gene expression by metastasis promoting protein S100A4 [32] and the regulation of optineurin promoter by NF-κB provide a basis for exploring the role of optineurin in cancer development.

On the basis of our results we suggest that there is a negative feedback loop in which TNFα-induced NF-κB activity induces expression of optineurin, which itself functions as a negative regulator of NF-κB. Some other negative regulators of NF-κB, such as IκB and CylD, are also induced by NF-κB in response to TNFα [22], [27]. Since optineurin protein level increases significantly after 18–24 hours of treatment with TNFα, it is likely that optineurin plays a more significant role in modulating NF-κB activity at a later stage.

In conclusion our results show that the induction of optineurin gene expression and promoter activation by TNFα is mediated by NF-κB through a binding site in the promoter. Our results also suggest that the relationship between NF-κB and optineurin is quite complex because NF-κB induces optineurin, which negatively regulates NF-κB activity. Since NF-κB and optineurin have been linked to glaucoma, their reciprocal regulation might have relevance to etiopathogenesis of glaucoma.

## Materials and Methods

### Cell culture and transfections

A549 and HeLa cells were maintained at 37°C in a CO2 incubator in Dulbecco's modified Eagle's medium supplemented with 10% fetal bovine serum. The transfections were carried out using Lipofectamine Plus reagent (Invitrogen, San Diego, CA, USA) according to the manufacturer's instructions. All the plasmids for transfection were prepared by using Qiagen columns (Hilden, Germany). Human TNFα (Sigma, St Louis, MO, USA or Calbiochem) was added wherever indicated at a final concentration of 10–20 ng/mL).

### RT-PCR (Reverse transcription-polymerase chain reaction)

Total RNA was isolated using the TRIzol reagent (Invitrogen). Semiquantitative RT-PCR was carried out essentially as described previously [33]. RNA was reverse transcribed using reagents from the first-strand cDNA synthesis kit (Invitrogen). Optineurin forward (5′-GCTGCAAATGGATGAAATGAAGCA-3′) and reverse primer (5′-CCGCTCGAGAGATCAACACTTAAATGATGCAATCC-3′) were used for the amplification of human optineurin cDNA by PCR. Real time PCR for optineurin gene expression was carried out using primers, forward 5′- GACACGTTACAGATTCACGTGA- 3′ and reverse, 5′- ACTGTGCCCGGCCTGTTTTC-3′. GAPDH was used as a control, forward 5′- TCATCCATGACAACTTTGGTATC- 3′ and reverse, 5′- AGGGATGACCTTGCCCACAGCCTTGGCA- 3′.

### Expression vectors and antibodies

Rabbit polyclonal optineurin antibody was from Abcam. NF-κB p65 antibody for supershift, IRF-1, IκB and tubulin antibodies were obtained from Santa Cruz Biotechnology (Santa Cruz, CA, USA); HRP conjugated anti-mouse and anti-rabbit antibodies were from Amersham Pharmacia Biotech (Piscataway, NJ, USA). Human optineurin expression plasmid and its E50K mutant have been described by us previously [25]. D474N mutation was created by PCR as described [25]. Plasmids for expressing p65 NF-κB and I-κB super repressor in which two serines are mutated [34] were provided by D. Karunagaran (Indian Institute of Technology, Chennai, India).

### Cloning of optineurin promoter, reporter plasmids and reporter assays

The optineurin promoter was cloned from human genomic DNA by utilizing the PCR. The primers used were: forward, Opt-F, 5′-CCGCTCGAGTCAGGCTTGGCCAGGCA3′ Opt-R, 5′-CCCAAGCTTCTGCCGCCCGGCTTGGCTT3′. The amplified promoter fragment of 1077 bp was cloned into the pMOSBlue vector (Amersham) and sequenced. The sequence of this promoter matched completely with that present in the database (Homo sapiens chromosome 10 genomic contig, reference assembly, Ref. NT_077569.2 HS10_77618 from nucleotide 7504122 to 7505198). Deletion construct (357 bp; named DP) was made from the full length promoter (FP) using forward primer 5′-CCGCTCGAGACGGACAGCGAGGGTGGGTA-3′ and same reverse primer as used for amplifying full length promoter. The optineurin promoter fragment was then excised by digestion with HindIII and XhoI, and subcloned into the pGL3-BASIC vector (Promega, Madison, WI, USA). NF-κB site in both the FP and DP were mutated by site directed mutagenesis with the primers, 5′-GGG CCC CTC AAA TTC CCC GGC G-3′ and 5′-CGC CGG GGA ATT TGA GGG GCC C-3′. The nucleotide sequence of all constructs was confirmed by automated DNA sequencing. Putative transcription factor binding sites were determined by using MatInspector from Genomatics Software. For determining promoter activity the cells grown in 24-well plates were transfected with 100 ng of the required pGL3 construct, 50 ng of pCMV.SPORT-β-gal (Invitrogen) and with the required amount of the other plasmids. The total amount of plasmid in each transfection was kept constant (400 ng for each well of a 24-well plate) by adding control plasmid. Lysates were generally made 24 hour post-transfection. Preparation of lysates and luciferase assays were carried out as per the instruction of manufacturer (Promega). Relative luciferase activities were calculated after normalizing with β-galactosidase enzyme activities.

### Western blot analysis

Cells were washed twice with PBS and lysed in 1× SDS sample buffer. Proteins were separated on 10% SDS-polyacrylamide gels and blotted onto nitrocellulose membranes, and processed further for western blotting as described [33].

### Electrophoretic Mobility Shift Assay (EMSA)

EMSA was carried out as previously described in detail [35]. Briefly, nuclear extracts were prepared from A549 cells after treatment with TNF-α for 15 min and then stored at −70°C. EMSA was performed by incubating 2 µl of nuclear extract for 15 min at 37°C with 1 ng of ^32^P-end-labeled 22-mer double-stranded oligonucleotide containing the NF-κB-binding site (5′-GGG CCC GGG AAA TTC CCC GGC G-3′). The DNA-protein complex formed was separated from free oligonucleotide on 5% native polyacrylamide gel, and the gel was then dried. The specificity of binding was examined by competition with the unlabeled self, mutant and consensus oligonucleotides.

### Generation of adenoviral vectors expressing shRNA

Downregulation of endogenous optineurin was achieved by shRNA (small hairpin RNA) mediated knockdown. For this purpose adenoviral vectors expressing shRNAs were generated. As a first step the shRNA expression vectors targeting two different regions of optineurin were generated essentially as described [33], [36]. The desired oligonucleotides were annealed and cloned into BbsI-XbaI sites of U6 promoter plasmid mU6 pro. The optineurin sequence targeted by shOptn1 was from nucleotides 1076–1094 and for shOptn2 was from 2046–2064 (GenBank accession NM_001008211). The sequences of the oligonucleotides used for cloning were:

for shOptn1,
5′-tttGCACGGCATCGTCTAAATAttcaagagaTATTTAGACAATGCCGTGttttt-3′ and
5′-ctagaaaaaGCACGGCATTGTCTAAATAtctcttgaaTATTTAGACGATGCCGTGC-3′,
For shOptn2,
5′-tttGGCTTACCTCGTTCAAAGAttcaagagaTCTTTGAACAAGGTAAGCCCttttt-3′ and
5′-ctagaaaaaGGGCTTACCTTGTTCAAAGAtctcttgaaTCTTTGAACGAGGTAAGCC-3′.

The adenoviral vectors expressing shRNAs were generated by using pAdEASY system [37] kindly provided by B. Vogelstein (Howard Hughes Medical Institute and Sidney Kimmel Comprehensive Cancer Center, The John Hopkins Medical Institutions, Baltimore, MD, USA). The transcriptional units of shOPTN1 and shOPTN2 shRNAs from the pMU6 vector were subcloned into the adenoviral vector as described previously [37], [38]. The shRNA cassettes were released along with the mU6 promoter by HindIII-XbaI digestion and cloned into pAdTrack plasmid upstream of CMV-GFP cassette. Recombinant plasmids were generated by homologous recombination in AdEasier cells. The recombinant adenoviral plasmids were linearized with PacI and transfected in HEK293T cells using Lipofectamine 2000 (Invitrogen). Recombinant viruses were collected after 15 days by lysing these HEK293T cells. A control virus was also made which expressed unrelated shRNA of the same length. All these adenoviruses express GFP to monitor the infection of cells.

Adenoviral vectors for expressing optineurin and its E50K mutant were prepared as described [38] using pAdEASY system [37].

### NF-κB reporter assays

The reporter plasmid NF-κB-luc containing five tandem consensus NF-κB binding sites upstream of a luciferase reporter gene was used for measuring NF-κB activity. HeLa cells grown in 24 well dishes were transfected with 25 ng of the NF-κB-luc plasmid, 50 ng of pCMV.SPORT-β-gal (Invitrogen) along with the required amount of other plasmids. The total amount of plasmid in each transfection was kept constant (400 ng for each well of a 24-well plate) by adding control plasmid. Lysates were made 26 h post-transfection.

### Indirect immunoflourescence and microscopy

For immunoflourescence staining HeLa cells grown on coverslips were transfected with required plasmids and 30 hrs after trasnfections, cells were treated with TNF-α for 30 min. Cells were then washed in PBS and fixed in 3.7% formaldehyde. Fixed cells were permeabilized and stained for p65 and optineurin by serially incubating with antibodies to p65 (Santa Cruz), anti rabbit-cy3 followed by mouse monoclonal HA (Roche) and anti mouse fluorescein isothiocyanate [39], [40]. The coverslips were then observed in a Olympus BX60 microscope and images captured using CoolSnap CCD camera.

### Statistical analysis

Graphs represent average ±SD values. Statistical differences were calculated using Student's T-test. When significant differences were observed, P values for pair wise comparisons were calculated by using two-tailed T-test. P values less than 0.05 was considered significant.
